# Distinct Mechanisms Regulate Lck Spatial Organization in Activated T Cells

**DOI:** 10.3389/fimmu.2016.00083

**Published:** 2016-03-08

**Authors:** Natasha Kapoor-Kaushik, Elizabeth Hinde, Ewoud B. Compeer, Yui Yamamoto, Felix Kraus, Zhengmin Yang, Jieqiong Lou, Sophie V. Pageon, Thibault Tabarin, Katharina Gaus, Jérémie Rossy

**Affiliations:** ^1^EMBL Australia Node in Single Molecule Science, School of Medical Science, University of New South Wales, Sydney, NSW, Australia; ^2^ARC Centre of Excellence in Advanced Molecular Imaging, University of New South Wales, Sydney, NSW, Australia

**Keywords:** Lck, T cell signaling, assembly of signaling complexes, membrane organization, super-resolution fluorescence microscopy, image correlation spectroscopy

## Abstract

Phosphorylation of the T cell receptor (TCR) by the kinase Lck is the first detectable signaling event upon antigen engagement. The distribution of Lck within the plasma membrane, its conformational state, kinase activity, and protein–protein interactions all contribute to determine how efficiently Lck phosphorylates the engaged TCR. Here, we used cross-correlation raster image correlation spectroscopy and photoactivated localization microscopy to identify two mechanisms of Lck clustering: an intrinsic mechanism of Lck clustering induced by locking Lck in its open conformation and an extrinsic mechanism of clustering controlled by the phosphorylation of tyrosine 192, which regulates the affinity of Lck SH2 domain. Both mechanisms of clustering were differently affected by the absence of the kinase Zap70 or the adaptor Lat. We further observed that the adaptor TSAd bound to and promoted the diffusion of Lck when it is phosphorylated on tyrosine 192. Our data suggest that while Lck open conformation drives aggregation and clustering, the spatial organization of Lck is further controlled by signaling events downstream of TCR phosphorylation.

## Introduction

T lymphocytes participate in an immune response when they become activated through the T cell receptor (TCR). However, despite the identification of the major players and sequences of events involved in T cell signaling pathways, the question of “How does T cell receptor signaling begin?” remains poorly understood (1, 2). TCR signaling is initiated when peptides bound to major histocompatibility complexes (pMHC) engage the TCR. The first detectable signaling event is the phosphorylation of immunoreceptor tyrosine-based activation motifs (ITAMs) on TCR/CD3 subunits by the Src kinase Lck. Lck is attached to the plasma membrane through the myristoylation and palmitoylation of residues at its amino terminus. Next to the membrane anchor are a Src homology 3 (SH3) and a SH2 domains, followed by a catalytic tyrosine kinase domain and a short carboxy-terminal tail. Phosphorylation and dephosphorylation of a carboxy-terminal inhibitory tyrosine (Y505) and an activating tyrosine (Y394) in the catalytic domain regulate Lck kinase activity. Lck activity is directly linked to its conformation, as phosphorylated Y505 binds intramolecularly to the SH2 domain, thereby promoting a closed state that prevents substrate access to the kinase domain. A large percentage of Lck is already phosphorylated on Y394 in resting cells and the proportion of active Lck is not dramatically increased upon TCR activation (3), although the opening of Lck is locally promoted at TCR engagement sites (4). To phosphorylate the TCR, the kinase and substrate must be in close proximity in order to interact, initiate, and sustain signaling; yet the underlying mechanisms for this molecular process are unknown.

The spatial organization of Lck is regulated by several different mechanisms. Lck can bind to and diffuse with the coreceptor CD4, which in turn binds to the pMHC complex on the antigen-presenting cell (1, 2). This association is thought to deliver Lck to the TCR and facilitate the phosphorylation of intracellular domains on the TCR–CD3 complex by Lck (5–7). The role of CD4 in facilitating TCR phosphorylation by Lck is ambiguous and complex. Indeed, while TCR signaling can occur in the absence of coreceptors (8, 9), CD4 association with Lck seems to be crucial for MHC restriction during thymic selection. The initial recruitment model was proposed based on the observations of Xu and Littman, in which initial TCR phosphorylation is mediated by coreceptor-independent Lck, while the coreceptor recruitment to TCR–CD3 complex occurs in a subsequent step (10). More recent studies support a model, in which CD4 sequesters most of the Lck molecules, thereby limiting the pool of Lck available to phosphorylate TCR that have not engaged a MHC molecules (11, 12). However, the work of Stepanek et al. suggest that only very few CD4 molecules are coupled to Lck and that TCR scans multiple CD4 to find one that is coupled to Lck (13).

The SH2 and SH3 domains of Lck mediate intramolecular interactions and the binding to a great variety of signaling proteins, such as TCRζ, Zap70, Csk, and CD45, as well as adaptor proteins, such as LIME and TSAd (14). These interactions may potentially modulate Lck diffusion or distribution within the membrane. Diffusing Lck can also be trapped in protein microdomains (15). We have previously shown that TCR activation triggers the clustering of Lck. Interestingly, this clustering was controlled by the conformation of the kinase, with the open/active form inducing clustering and the inactive/closed form preventing it, thereby establishing a link between the distribution of Lck and its kinase activity (16).

Our previous results suggest a relationship between the clustering of Lck and a local increase in signaling ability. Because T cells tightly regulate the strength and extent of TCR signaling, it is likely that the molecular processes following TCR activation impact on Lck distribution, thereby retroactively modulating Lck activity. Such a feedback mechanism has already been shown for the regulation of Zap70 clustering by SLP-76 (17). The SH2 domain of Lck represents a privileged candidate to facilitate such a feedback mechanism. Indeed, not only does this single binding domain connect Lck to a great variety of adaptors and effectors, more importantly, its binding affinity is regulated by phosphorylation on an adjacent tyrosine 192 (Y192). Activation of TCR triggers phosphorylation on Y192 (18, 19), which modifies the binding of the SH2 domain to Lck substrates and correlates to reduced signaling downstream of the TCR (20, 21). Thus, phosphorylation on Y192 can induce a switch in Lck-binding partners and may also affect the distribution of the kinase in the membrane.

Two recent studies illustrate the role of Y192 in regulating Lck activity and interactions. First, Granum et al. showed that preventing phosphorylation of Y192 (Lck Y192F mutation) led to a greater extent of tyrosine phosphorylation, including CD3ζ. This study also identified various proteins, including the adaptor protein TSAd, which displayed a greater affinity for the SH2 domain of Lck when Y192 was phosphorylated (21). TCR activation promotes the phosphorylation of TSAd by Lck as well as their association, which potentially inhibits Lck activity (22–24) and further enhances Y192 phosphorylation (21).

The second study by Sjölin-Goodfellow et al. demonstrated that selective inhibition of the kinase Zap70 led to a pronounced decrease in Y192 phosphorylation on Lck in resting and activated cells. This coincided with an increased phosphorylation of the Lck-activating tyrosine 394 (Y394) (25). Lck and Zap70 functions and activities are tightly intertwined in T cell signaling, making Zap70 another likely candidate for regulating Lck distribution. Zap70 binds to the intracellular ITAM domains of the TCR complex after they are phosphorylated by Lck. Finally, Lck further binds to the phosphorylated tyrosine 319 on Zap70, an event that stabilizes the activated conformation of Lck and facilitates the activation of Zap70 (26). Interestingly, Zap70 inhibition does not affect Lck phosphorylation on the activating Y394 (27), suggesting that if Zap70 can regulate Lck activity, it is likely to do so through the control of Lck localization. Once recruited to the ITAMs of TCR and fully activated, Zap70 phosphorylates the adaptor protein Lat. Lat too is susceptible of modifying Lck distribution, as it interacts with Lck upon TCR activation (28, 29) and preferentially associates with the open form of Lck (30). Lat also contributes to Lck phosphorylation at Y394 upon TCR stimulation (29).

Hence, we set out to quantify the contribution of the phosphorylation on Y192 Lck, Zap70, and Lat to the spatial organization of Lck in activated Jurkat T cells using cross-correlation raster image correlation spectroscopy (ccRICS) and photoactivated localization microscopy (PALM). We used an open mutant, Lck(Y505F), as well as a mutant that cannot be phosphorylated on Y192, Lck(Y192F), and measured their diffusion and propensity to aggregate and cluster in Lck-deficient Jurkat T cells reconstituted with Lck and in Jurkats lacking Zap70 or Lat. Our results show that similarly to locking Lck in an open conformation, preventing phosphorylation on Y192 promotes Lck clustering, albeit *via* a fundamentally different mechanism. While clustering of open Lck was found to be intrinsic and only attenuated by the absence of Zap70 and Lat, clustering of Lck(Y192F) was not associated with self-aggregation and was dramatically modified in cells lacking Zap70 or Lat. These data suggest that while Lck open conformation drives aggregation and clustering, Lck spatial organization is further controlled by signaling events happening downstream of TCR ITAMs phosphorylation.

## Results

### Interaction of Diffusing Proteins in Jurkat T Cells Measured with ccRICS

We used raster image correlation spectroscopy (RICS) to extend the data on Lck spatial organization obtained previously in fixed and live cells using PALM (16). The RICS method derives information on protein diffusion and binding dynamics in live cells by spatiotemporal correlation analysis of fluctuations in fluorescence intensity acquired within the pixels of a time series of images (31). If the acquisition is extended to a two-color experiment, then a ccRICS analysis can be carried out between the two channels to extract the fraction of interacting molecules, based on the principle that proteins moving as part of the same complex will give rise to fluctuations in fluorescence intensity that positively cross correlate (32).

**Figure 1:**
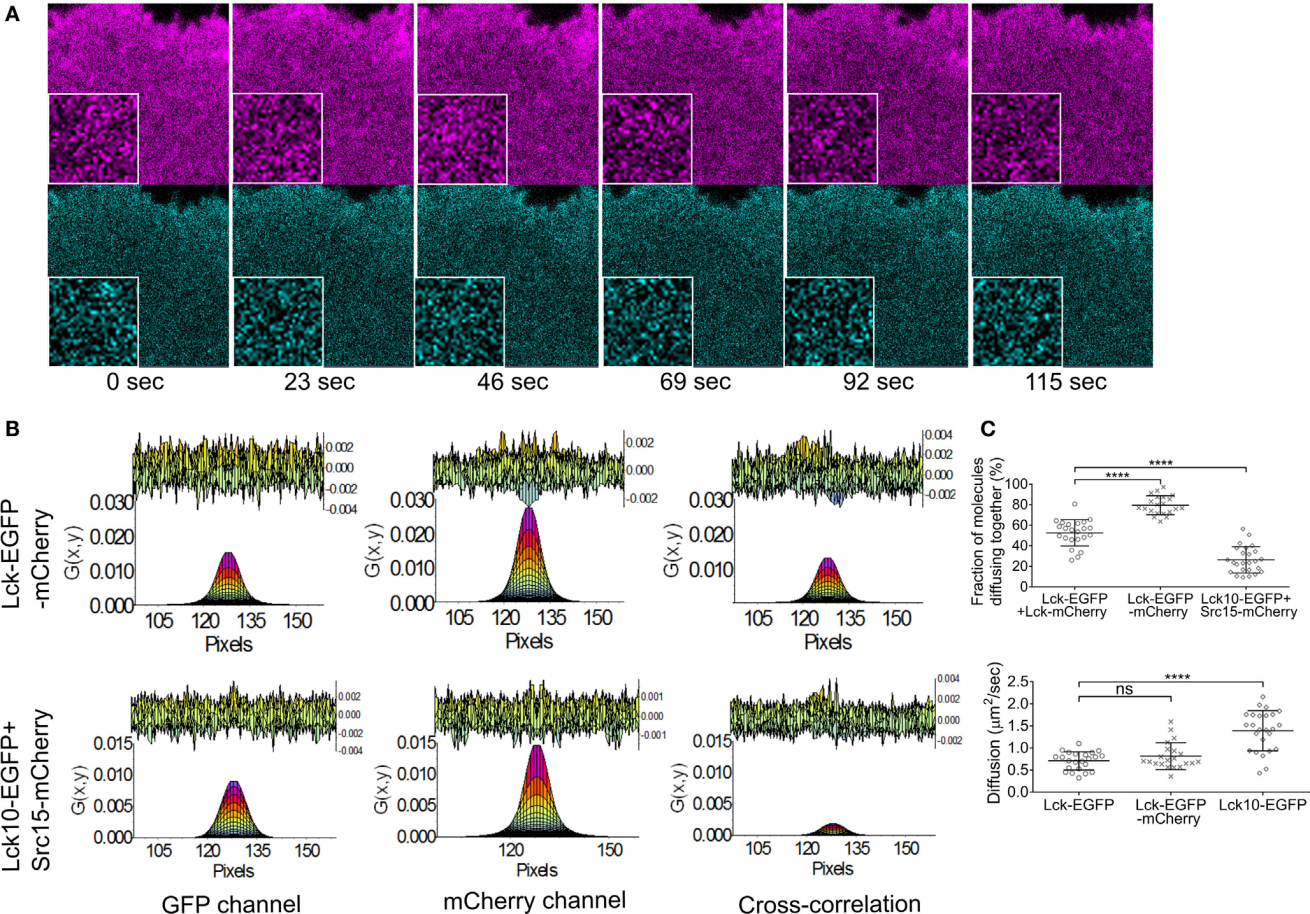
**Validity of the ccRICS approach to measure the interaction of diffusing proteins in Jurkat T cells**. JCaM1 cells transfected, respectively, with (1) WT Lck–EGFP and WT Lck–mCherry, (2) Lck–EGFP–mCherry (positive control), and (3) Lck10–EGFP and Src15–mCherry (negative control) were pre-activated for 10 min on glass coverslips coated with activating anti-CD3 and anti-CD28, and then imaged for approximately 3 min (100 frames) between 10 and 40 min of activation. **(A)** Example of images used for ccRICS – cells transfected with Lck–EGFP–mCherry. Inset: zoom of a 32 × 32 pixels region. **(B)** One-component fitting of the RICS function in the EGFP and mCherry channels and the ccRICS function between the two channels for the positive (upper row) and negative controls (lower row). The top of the charts shows the residual component of the fit. **(C)** Top: fractions of molecules diffusing together. Bottom: diffusion coefficients for WT Lck, the positive control and the negative control extracted, respectively, from the cross-correlation and EGFP autocorrelation functions. Each symbol in **(B)** represents one cell; small horizontal lines indicate mean (±SEM). ns, not significant; **P < 0.005 and ****P < 0.0005 (unpaired *t*-test). Data are from three independent experiments with at least 21 cells.

To test the validity of this approach for studying Lck dynamic and interactions in activated Lck-deficient Jurkat cells – JCaM1 – we transiently expressed or co-expressed the following constructs: Lck–EGFP and Lck–mCherry, Lck–EGFP–mCherry, and Lck10–EGFP and Src15 mCherry, i.e., the two unrelated membrane anchors of Lck and Src, respectively. Cells were activated on coverslips coated with antibodies against CD3ϵ and CD28 and imaged between 10 and 40 min of activation as described in Section “Materials and Methods.” We quantified the percentage of EGFP and mCherry proteins diffusing together from the amplitude of the ccRICS function, which is a measure of their interaction (Figure 1A). About half of the Lck molecules were interacting when the two fluorescent proteins were on separate copies of Lck (52 ± 13%), while 80 ± 10% of EGFP and mCherry diffused together when the fluorescent proteins were fused together in the positive control Lck–EGFP–mCherry. By contrast, only 26 ± 15% of Lck10 molecules were associated with Src15 (Figure 1B). These results confirm that ccRICS identified the EGFP and mCherry labels attached to the same Lck molecules as diffusing together and two unrelated membrane anchors as predominantly not associated. The diffusion coefficients were as expected similar for the single- or double-labeled Lck, while Lck10 diffused significantly faster (0.7 ± 0.2, 0.8 ± 0.2, and 1.4 ± 0.5 μm^2^/s, respectively). These values reflected the fact that Lck10 only contained the membrane anchor that cannot interact with other proteins while full-length Lck is larger and has the potential for protein–protein interactions. These values are also within the same range than those previously measured by single particle tracking (15, 33, 34).

Together these data demonstrate that approximately half of the population of Lck molecules interacts with each other in activated Jurkat cells and that ccRICS is a suitable methodology to measure Lck interactions and diffusion.

### Lck Clustering Was Facilitated by Two Distinct Mechanisms

To compare the contribution of two different mechanisms – conformational state of Lck versus protein–protein interactions mediated by the SH2 domain of Lck upon TCR activation – to the spatial distribution of Lck, we expressed WT Lck, Lck(Y505F), and Lck(Y192F), fused to either EGFP or mCherry in the Lck-deficient Jurkat T cell line JCaM1. Lck(Y505F) cannot be phosphorylated on the inhibitory Y505 and is therefore locked into an open conformation. When Lck cannot be phosphorylated on Y192, its SH2 domain is prevented from binding to many potential interactors, including the kinases Pyk2 and Itk, the phosphatase SHP-1, and the adaptor protein TSAd (21). We co-expressed each Lck variant labeled with EGFP and with mCherry to investigate their self-association. As for Figure 1, live cells were imaged between 10 and 40 min of activation on glass coverslips coated with antibodies against CD3ϵ and CD28.

**Figure 2:**
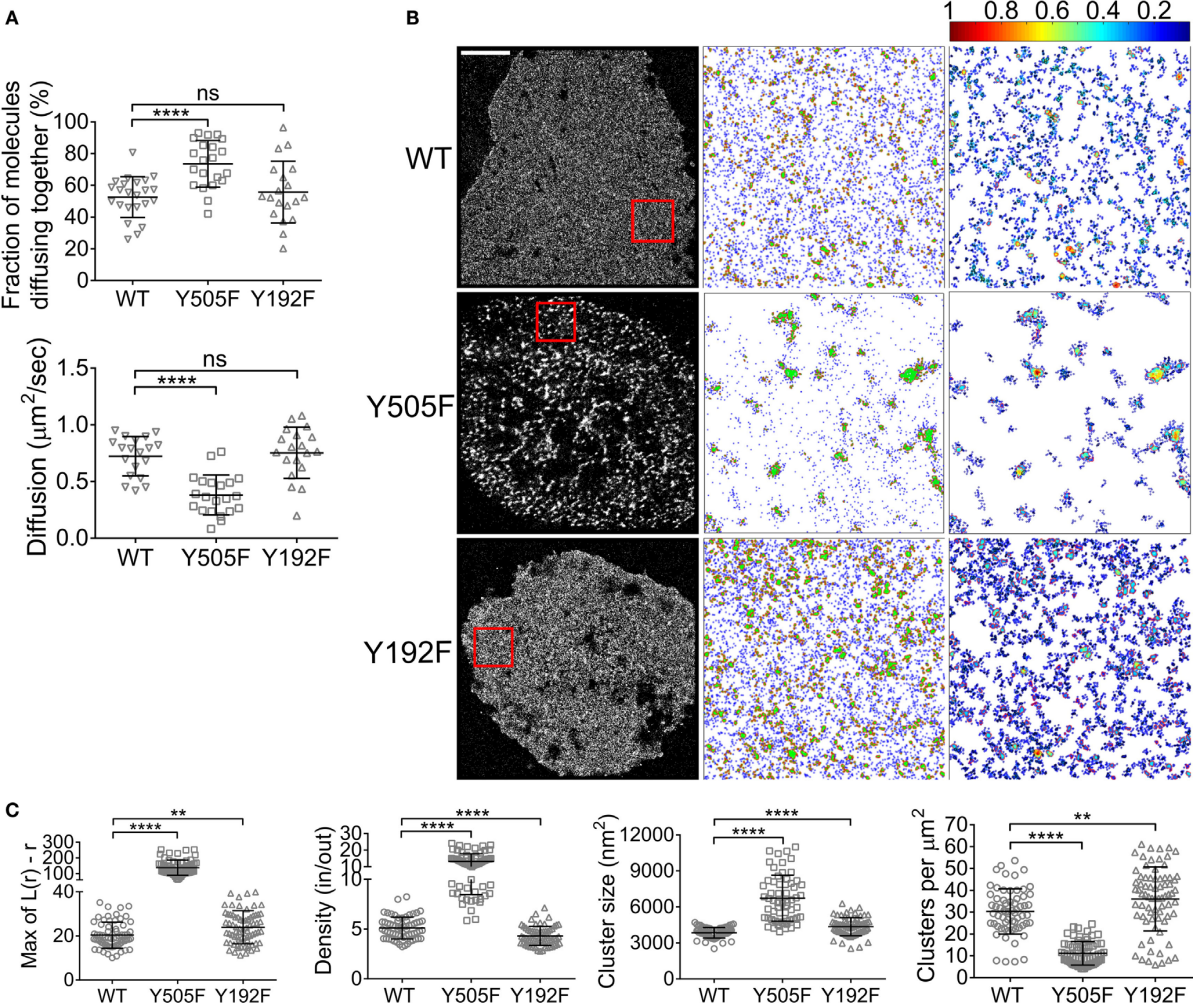
**Intrinsic and SH2-mediated Lck clustering**. **(A)** JCaM1 cells expressing (1) WT Lck–EGFP and WT Lck–mCherry, (2) constitutively open Lck(Y505F)–EGFP and Lck(Y505F)–mCherry, or (3) SH2-binding mutant Lck(Y192F)–EGFP and Lck(Y192F)–mCherry were activated and imaged as in Figure 1. Top: fractions of molecules diffusing together. Bottom: diffusion coefficients for WT Lck, open Lck, and the SH2 Lck mutant. Data for WT Lck are the same than data plotted in Figure 1. **(B)** First column: single-molecule PALM images of WT Lck–PS-CFP2, Lck(Y505F)–PS-CFP2, and Lck(Y192F)–PS-CFP2 in JCaM1 cells incubated on glass coverslips coated with activating anti-CD3 and anti-CD28 and fixed after 10 min. Scale bars, 5 μm. Middle column: cluster maps generated by DBSCAN analysis from the 4 μm × 4 μm regions highlighted in red. Last column: maps showing the clusters identified by DBSCAN and color-coded for relative density (0–1). **(C)** From left to right: maxima (Max) of Ripley’s *K* function curves of image regions and relative density in clusters, cluster size, and number per area obtained from DBSCAN analysis. Each symbol represents one cell **(A)** or one image region **(C)**; small horizontal lines indicate mean (±SEM). ns, not significant; **P < 0.001 and ****P < 0.00005, unpaired *t*-test for the ccRICS data **(A)** and Mann–Whitney test for the PALM data **(C)**. Data are from three to five independent experiments with a total of at least 19 cells.

Around 74% of Lck(Y505F) and 55% of Lck(Y192F) were found to self-associate (Figure 2A), which corresponds to the values for the EGFP–mCherry positive control and WT Lck, respectively (Figure 1). The high propensity of Lck(Y505F) to self-aggregate was also reflected in a low diffusion coefficient that was of about half that of WT Lck (0.38 ± 0.1 versus 0.7 ± 0.2 μm^2^/s, respectively). Interestingly, the tendency of Lck(Y505F)–EGFP to diffuse with WT Lck–mCherry was similar to that measured for WT Lck, suggesting that open Lck only interacts with open Lck and does not recruit WT Lck into clusters of open Lck (Figure S1 in Supplementary Material). In parallel to the ccRICS experiments, we expressed WT Lck, Lck(Y505F), and Lck(Y192F) fused to the photo-switchable fluorescent protein PS-CFP2 in JCaM1 cells and imaged them after 10 min of activation using PALM. As previously described (16), the open Lck mutant Lck(Y505F) displayed a very high level of clustering [quantified by the Ripley *K* function, *L*(*r*) − *r*], assembling in clusters that were denser, larger, and less numerous than WT Lck (Figures 2B,C). Lck(Y192F) also clustered significantly more than WT Lck, albeit in a very different way than Lck(Y505F), forming more clusters of lower density (Figures 2B,C). Level of clustering of WT Lck and Lck(Y192F) were similar in resting cells (Figure S1B in Supplementary Material). Finally, expression of CD4 in JCaM1, which do not express endogenous CD4, did not have a significant impact on either Lck diffusion or clustering (Figure S2 in Supplementary Material).

These data point toward two different mechanisms for the clustering of the constitutively open versus the low-affinity SH2 mutants of Lck. On the one hand, locking Lck in the open conformation intrinsically increased its affinity for itself, as illustrated by ccRICS, which led to a low number of very dense clusters. On the other hand, preventing the TCR activation-induced affinity of Lck SH2 domain did not affect its affinity for self but somehow unexpectedly led to the formation of many clusters of low density. This suggests that the interactions mediated by the Lck SH2 domain when Y192 is phosphorylated somehow prevents the close packing of Lck in clusters and correlates to the inhibitory effect of Y192 phosphorylation on TCR signaling (20, 21).

### Zap70 Enhanced the Clustering of Lck(Y505F) and Was Required for Lck(Y192F) Clustering

**Figure 3:**
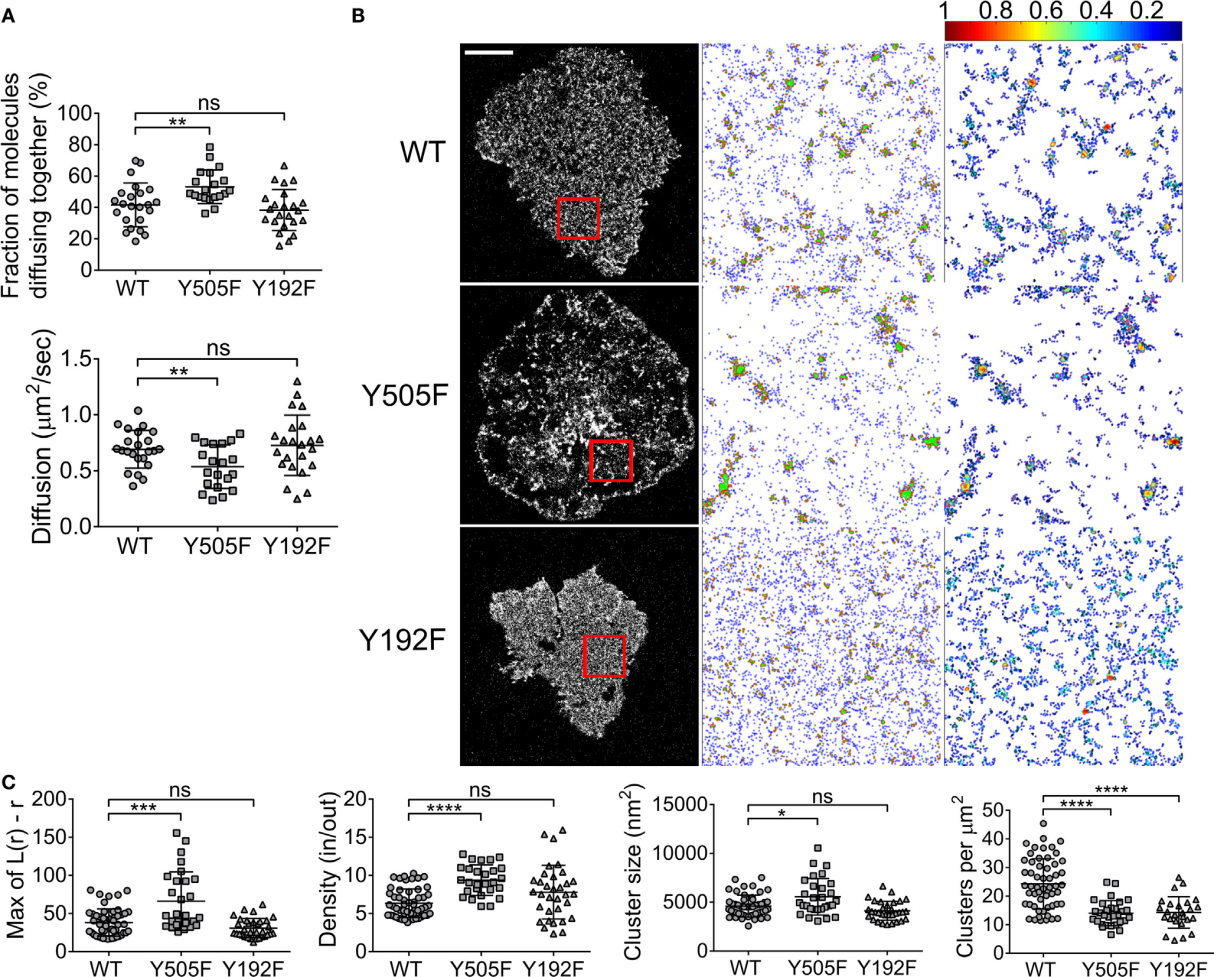
**Zap70 promotes intrinsic clustering of open Lck and is required for clustering of SH2 mutant Lck**. **(A)** Zap70-deficient P116 cells expressing (1) WT Lck–EGFP and WT Lck–mCherry, (2) constitutively open Lck(Y505F)–EGFP and Lck(Y505F)–mCherry, or (3) SH2-binding mutant Lck(Y192F)–EGFP and Lck(Y192F)–mCherry were activated and imaged as in Figure 1. Top: fractions of molecules diffusing together. Bottom: diffusion coefficients for WT Lck, open Lck, and the SH2 Lck mutant. **(B)** First column: single-molecule PALM images of WT Lck–PS-CFP2, Lck(Y505F)–PS-CFP2, and Lck(Y192F)–PS-CFP2 in P116 cells incubated on glass coverslips coated with activating anti-CD3 and anti-CD28 and fixed after 10 min. Scale bars, 5 μm. Middle column: cluster maps generated by DBSCAN analysis from the 4 μm × 4 μm regions highlighted in red. Last column: maps showing the clusters identified by DBSCAN and color-coded for relative density (0–1). **(C)** From left to right: maxima (Max) of Ripley’s *K* function curves of image regions and relative density in clusters, cluster size, and number per area obtained from DBSCAN analysis. Each symbol represents one cell **(A)** or one image region **(C)**; small horizontal lines indicate mean (±SEM). ns, not significant; *P < 0.05, ***P < 0.0005, and ****P < 0.00005, unpaired *t*-test for the ccRICS data **(A)** and Mann–Whitney test for the PALM data **(C)**. Data are from three independent experiments with a total of at least 20 cells.

Zap70 is essential for T cell signaling, acting immediately downstream of Lck. Zap70 can bind to the SH2 domain of Lck (26) and is essential for Y192 phosphorylation on Lck (25). Hence, we repeated the ccRICS and PALM measurements of WT Lck, Lck(Y505F), and Lck(Y192F) in a Zap70-deficient Jurkat T cell line, P116, in order to determine how the absence of Zap70 impacts on the spatial organization of Lck. Locking Lck in an open conformation still promoted Lck self-aggregation compared to WT Lck, albeit to a much lower extent than in JCaM1 cells (from 41 ± 9 to 53 ± 14%). It is possible that the presence of endogenous unlabeled open Lck reduced the detected fraction of co-diffusing Lck molecules. As in JCaM1 cells, we observed no difference in self-aggregation for Lck(Y192F) relative to WT Lck (Figure 3A). Diffusion of Lck(Y505F) was decreased to the same extent than self-association and diffusion of Lck(Y192F) was not affected (Figure 3A). The increase of Lck(Y505F) clustering compared to WT Lck as measured by PALM followed the trend observed in the ccRICS measurement, displaying the same but attenuated changes in density, size, and number of clusters in P116 cells as in JCaM1 cells (Figures 3B,C). In contrast, absence of Zap70 did not have any significant effect on Lck(Y192F) clustering compared to WT Lck (Figures 3B,C). Finally, the comparison of WT Lck clustering in JCaM1 cells and in P116 cells revealed that the absence of Zap70 clearly promote Lck clustering trough a mechanism that does not rely on self-association (Figure S3 in Supplementary Material). However, this observation has to be moderated by the fact that JCaM1 and P116 cells may have different homeostasis to compensate for the lack of Lck and Zap70, respectively.

These data suggest that Zap70 was differentially involved in the two type of Lck clustering as we observed in Figure 2: Zap70 only had modest impact on conformation-induced clustering but severely impacted on Lck clustering facilitated by the high affinity state of the Lck SH2 domain. Because of the differential effect, the data support the idea that two distinct mechanisms exist for Lck clustering.

### Lat Contributed to the Clustering of Open Lck But Repressed Lck(Y192F) Clustering

**Figure 4:**
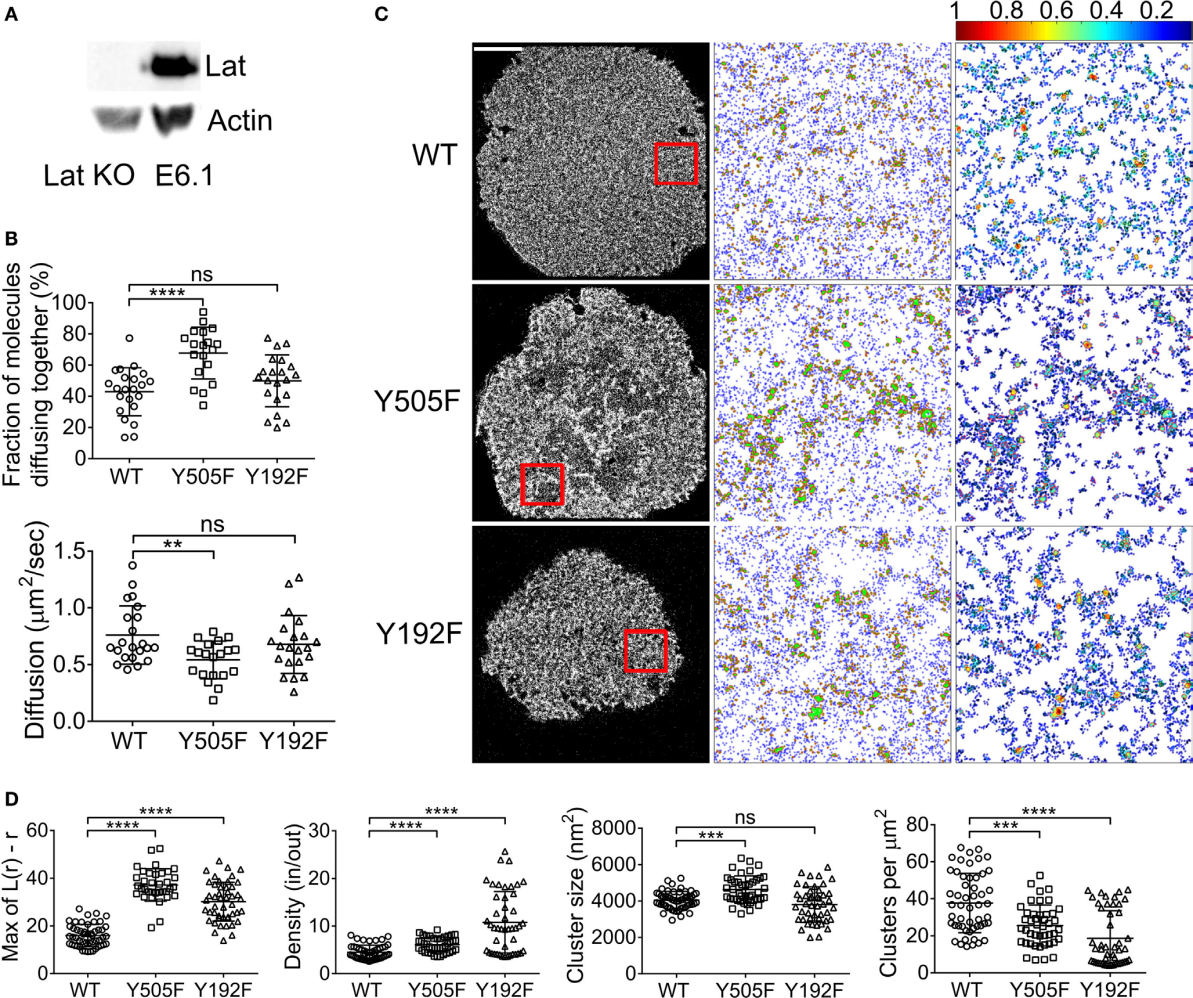
**Lat contributes to the clustering of open Lck but represses the clustering of the SH2 mutant Lck**. **(A)** Immunoblot of Lat KO cells and wild-type E6.1 Jurkat cells. **(B)** Lat KO cells expressing (1) WT Lck–EGFP and WT Lck–mCherry, (2) Lck(Y505F)EGFP and Lck(Y505F)–mCherry, or (3) Lck(Y192F)–EGFP and Lck(Y192F)–mCherry were activated and imaged as in Figure 1. Top: fractions of molecules diffusing together. Bottom: diffusion coefficients for WT Lck, open Lck, and the SH2 Lck mutant. **(C)** First column: single-molecule PALM images of WT Lck–PS-CFP2, Lck(Y505F)–PS-CFP2, and Lck(Y192F)–PS-CFP2 in Lat KO cells incubated on glass coverslips coated with activating anti-CD3 and anti-CD28 and fixed after 10 min. Scale bars, 5 μm. Middle column: cluster maps generated by DBSCAN analysis from the 4 μm × 4 μm regions highlighted in red. Last column: maps showing the clusters identified by DBSCAN and color-coded for relative density (0–1). **(D)** From left to right: maxima (Max) of Ripley’s *K* function curves of image regions and relative density in clusters, cluster size, and number per area obtained from DBSCAN analysis. Each symbol represents one cell **(A)** or one image region **(B)**; small horizontal lines indicate mean (±SEM). ns, not significant; **P < 0.005, ***P < 0.0005, and ****P < 0.00005, unpaired *t*-test for the ccRICS data **(B)** and Mann–Whitney test for the PALM data **(D)**. Data are from three to five independent experiments with a total of at least 20 cells.

In the canonical model of the TCR signaling cascade, the primary target of Zap70 kinase activity is the adaptor protein Lat (1, 26). It has also been demonstrated that Lat interacts with Lck with a predilection for the open conformation (28–30). In order to evaluate whether Lat influences Lck spatial organization, we performed the same ccRICS and PALM experiments in Jurkat T cells where Lat expression had been knocked out with CRISPR/Cas9 gene editing (Figure 4A). In contrast to what our observation in Zap70-deficient cells (Figure 3A), the intrinsic tendency of Lck(Y505F) to self-aggregate more than WT Lck was similar to what we measured in cells expressing Lat (Figure 4B, 68 ± 16 and 43 ± 15%, respectively). The diffusion coefficient of open Lck was also decreased compared to WT Lck (Figure 4B, 0.76 ± 0.2 and 0.54 ± 0.2 μm^2^/s, respectively), although to a slightly lower extent than what we observed in the presence of Lat. Lck(Y192F) self-aggregation and diffusion coefficient were not affected by the absence of Lat (Figure 4B).

Similarly to the ccRICS data, the PALM data showed that open Lck(Y505F) was more clustered than WT Lck in Lat-deficient cells, although to a much lesser extent to what we observed in cells expressing Lat (Figures 4C,D). More interestingly, knocking out Lat boosted the SH2-related clustering of Lck(Y192F), mostly by drastically increasing the density of Lck(Y192F) clusters (Figures 4C,D). Comparing WT Lck clustering in Lat-deficient cells and JCaM1 showed that Lat promotes Lck clustering of WT Lck and Lck(Y505F) (Figure S3A in Supplementary Material). Bypassing Lat signaling by stimulating Lat KO cells with PMA + ionomycin did return clustering levels of WT Lck to the values observed in cells expressing Lat, but not of Lck(Y505F), suggesting that Lat might regulate the clustering of WT and open Lck through different mechanisms or that clustering of WT Lck is more sensitive to the ionic strength of the cytoplasm (Figure S3B in Supplementary Material).

In light of these results, it appears that Lck(Y192F) was allowed to “cluster freely” even more in the absence of Lat, suggesting that the protein network organized by Lat contributes to restraining Lck clustering *via* SH2 domain interacting partners. Additionally, Lck(Y505F) clustered less in absence of Lat and Zap70; however, the potentiating effects of Zap70 on the clustering of open Lck were far greater than those of Lat on open Lck clustering. This is in agreement with a TCR network topology, where Zap70 is more closely located to Lck than Lat.

### TSAd Bound to Lck and Promoted Its Diffusion

**Figure 5:**
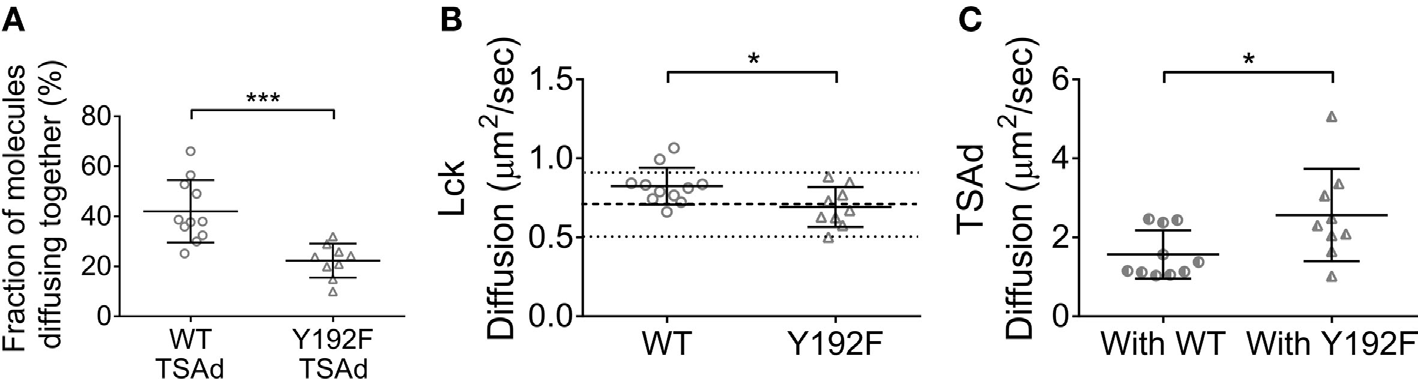
**TSAd binding to Lck promotes Lck diffusion**. JCaM1 cells transfected, respectively, with (1) WT Lck–EGFP and TSAd–mCherry, or (2) Lck(Y192F)–EGFP and TSAd–mCherry were activated and imaged as in Figure 1. **(A)** Fraction of TSAd–mCherry molecules diffusing together with either WT Lck–EGFP or Lck(Y192F)–EGFP. **(B)** Diffusion coefficients for WT Lck–EGFP and Lck(Y192F) in cells expressing TSAd–mCherry. **(C)** Diffusion coefficients for TSAd–mCherry in cells expressing WT Lck–EGFP or Lck(Y192F)–EGFP. Each symbol represents one cell; small horizontal lines indicate mean (±SEM). *P < 0.05 and ***P < 0.0005, unpaired *t*-test. Data are from three independent experiments with at least nine cells.

It was unexpected that preventing the TCR-dependent increase of the affinity of the Lck SH2 domain in the Lck(Y192F) mutant enhanced its clustering. It is logical to assume that the association of Lck with a protein network would rather immobilize Lck and promote cluster formation. In an attempt to understand this apparent contradiction, we focused on the adaptor protein TSAd, which was recently shown to associate with Lck upon Y192 phosphorylation (21). Lck–EGFP or Lck(Y192F)–EGFP were transiently co-expressed in JCaM1 cells together with TSAd–mCherry. Cells were imaged by ccRICS as for the Lck aggregation experiments to measure the association of Lck or Lck(Y192F) with TSAd. In accordance with the data of Granum et al., we observed that the fraction of Lck WT associating with TSAd was almost twofold higher than for Lck(Y192F) (Figure 5, 42 ± 12 and 22 ± 6%, respectively). Importantly, the diffusion measurements revealed that (a) the diffusion coefficient of WT Lck was increased in cells overexpressing TSAd, (b) but the diffusion coefficient of Lck(Y192F) was not affected by TSAd overexpression. The latter had a similar diffusion coefficient in these cells as WT Lck had in JCaM1 cells (Figure 5). The diffusion of TSAd followed the exact opposite trend, being slower when Lck could bind to TSAd and faster for the Lck(Y192F) mutant. These data suggest that upon phosphorylation of Y192, Lck bound to and co-diffused with the fast moving TSAd, which consequently decreases the probability of Lck to be immobilized in clusters.

## Discussion

We have demonstrated previously that TCR activation leads to an increase in Lck clustering and that this clustering is driven by the open/active conformation of Lck (16). Here, we confirmed that open Lck has an intrinsic tendency to assemble into clusters, as it displayed a higher affinity for self and diffused slower than WT Lck. Our data further indicated that signaling proteins downstream of TCR contributed to regulating Lck distribution in the plasma membrane of activated T cells, however, through mechanisms that were not related to conformation-induced clustering. Indeed, preventing the phosphorylation of Y192 by a tyrosine to phenylalanine point mutation (Y192F) also resulted in a significant increase in Lck clustering. Phosphorylation on Y192 is triggered by TCR activation (18, 19) and is associated with the downregulation of TCR signaling (20, 21). Functionally, Y192 phosphorylation represents a signaling switch that controls the affinity of the Lck SH2 domain for tyrosine-phosphorylated-interacting partners. The phosphatase SHP-1 is among the proteins that display a greater affinity for Lck upon Y192 phosphorylation (21) and is at the same time the central element of a negative feedback mechanism that dephosphorylates Lck and TCR upon TCR activation (35). One could thus speculate that the Y192-mediated SHP-1 deactivation of Lck favors the closed conformation and consequently prevents Lck clustering. Preventing Y192 phosphorylation would then lead to more activated Lck and in turn enhance conformation-induced clustering. However, we found identical levels of self-association for Lck(Y192F) and WT Lck with ccRICS and Lck(Y192F) clusters had very different characteristics than the Lck(Y505F) clusters when measured with PALM, being in much higher number and having a very low density. Hence, the clustering that we observed when we prevented phosphorylation of Y192 was fundamentally different from that of Lck(Y505F)-induced clustering and was more likely to be related to direct modifications of Lck spatial organization through protein–protein interactions mediated by its SH2 domain. Note that despite the differences in their cluster properties, Lck clustering induced by Y505F and Y192F correlate to an increased phosphorylation of the TCR (21, 36), suggesting that Lck is more efficient at phosphorylating TCR when in clusters.

In cells lacking Zap70, conformation-induced clustering of Lck was significantly attenuated compared to what we observed in JCaM1 cells. If we put the possible contribution of untagged open Lck in these cells aside, this suggests that Zap70 further promotes intrinsic clustering of open Lck. This could be achieved by favoring the confinement of Lck. Indeed, Zap70 kinase activity is essential to the assembly of the protein network downstream of Lat, which in turn is directly linked to actin regulation at the immunological synapse. Interactions both with the protein network installed by Lat and with the actin cytoskeleton could regulate Lck confinement. Zap70 can also have a kinase-independent scaffolding function (27), which could contribute to regulating Lck distribution through the direct binding of Lck to Zap70 (26). The picture gets even more complex when considering (a) that the absence of Zap70 in P116 cells reversed Y192F-induced changes in clustering, despite the fact that Y192 phosphorylation does not modify the affinity of Lck for Zap70 (21) and (b) that WT Lck clustering was greatly increased in P116 cells relative to cells expressing Zap70. All in all, the intricate relationship between Zap70 and Lck spatial distribution likely reflects the versatile role played by Zap70 in T cell signaling. Indeed, while Zap70 is essential for the propagation of TCR signaling (26) as a kinase and as an adaptor protein (27), it also mediates a negative feedback signaling that directly moderates Lck activity (25).

Lat interacts with Lck (28) and this interaction could be involved in the contribution of Lat to Lck(Y505F) clustering. However, this interaction cannot explain the link between Lat and Lck(Y192F) clustering as it is not mediated by an SH2 domain–phosphotyrosine association. Additionally, Y192 phosphorylation does not modify the affinity of Lck for Lat (21). It has been shown recently that preventing tyrosine phosphorylation on SLP-76, a scaffold protein downstream of Lat in the TCR signaling cascade, led to constitutively increased phosphorylation of Y192. On the other hand, knock-out of SLP-76 led to a constitutive decrease in Y192 phosphorylation (37). This suggests that the protein network assembled by Lat is susceptible of regulating Lck distribution through Y192 phosphorylation. Interestingly, while interactions with Lat recruit SLP-76 to the membrane and TCR complex, SLP-76 is phosphorylated by Zap70 (26, 38). Thus, the opposite effects we observed in Zap70- and Lat-deficient cells on the clustering of Lck(Y192F) could relate either to the lack of phosphorylation of SLP-76 tyrosine – in Zap70-deficient cells – or to the reduced recruitment of SLP-76 to the plasma membrane – in Lat-deficient cells.

We further observed that upon TCR activation, Y192 phosphorylation contributes to promoting Lck association with TSAd, an adaptor protein lacking enzymatic activity (21–23). There is conflicting evidence on the role of TSAd in T cell signaling (39), as knock-out of TSAd (22, 40) or its overexpression (23, 41) both impair T cell activation. However, TSAd-deficient mice had a higher susceptibility to T-cell-related autoimmune diseases (39), which rather supports the hypothesis of a moderating role for TSAd in T cell signaling. Our data showed that diffusing Lck and TSAd associated when Y192 could be phosphorylated and that this association was impaired in the Lck(Y192F) mutant. Given the fast diffusion of TSAd, it is possible that this SH2 domain-mediated association of Lck prevents the formation of dense Lck clusters and consequently downregulates Lck activity. Interestingly, Lat also interacts with TSAd (39), which could potentially favor the recruitment of TSAd to the plasma membrane. In this context, the absence of Lat might result in a lower probability of TSAd binding to Lck and explain why Lck(Y192F) clustering is boosted in Lat-deficient cells.

It has been previously suggested that binding to TSAd would promote the open conformation of Lck by breaking the SH2–pY505 intramolecular interaction (42). However, our data do not support this model, as the reduced association of TSAd and Lck(Y192F) versus WT Lck that we observed in ccRICS correlates to a higher level of clustering of Lck(Y192F) versus WT Lck. Nevertheless, the affinity of Lck SH2 domain for pY505 is indeed relatively low (43), and it is generally assumed that an engaged SH2 domain would promote the open conformation of Lck. In that respect, it would be very interesting to determine if phosphorylation on Y192 affects the affinity of SH2 domain for Y505, thereby establishing a link between the two mechanisms observed in this study.

Finally, Couture et al. reported that when phosphorylated on Y192, Lck bound to much less proteins, although these proteins were not identified (20). Hence, we cannot exclude that the increase in clustering observed for Lck(Y192F) is the consequence of Lck being engaged in more protein–protein interactions.

In conclusion, when bearing in mind the inhibitory effect of Y912 phosphorylation, we could speculate that the “declustering” of Lck when Y192 is phosphorylated is a way of downregulating Lck signaling, similarly to what has been described for Zap70 clusters (17). It could also be a way of “recycling” the Lck population engaged in clusters, either in order to allow Lck molecules to search for more triggered TCRs or to allow Lck to engage in other processes related to later T cell signaling. For instance, once released from clusters, Lck molecules would be more likely to bind Itk for later signaling events as suggested previously (21).

## Materials and Methods

### Plasmids and CRISPR/Cas9

Mammalian expression constructs encoding full-length wild-type human Lck and the constitutively open Lck(Y505F) mutant were a gift from T. Harder. PS-CFP2 expression backbone was obtained from Evrogen. The Y192F single point-substitution mutants of Lck were made by site-directed mutagenesis. The 10- and 15-amino acid N-terminus regions of Lck and Src were fused to EGFP and mCherry, respectively, *via* a short 4 amino acid (GGGG) linker. Lck–EGFP–mCherry was made by cloning the mCherry coding sequence into pm-Lck–EGFP-N1 using *Age*I.

For the knocking out of Lat, Jurkat cells were transfected with two gRNAs (guide RNA) that were specifically designed to target genomic Lat DNA, together with cas9 expression plasmid. Twenty-four hours post-transfection, transfected single cells were FACS sorted and seeded into 96-well plates. Cell clones were screened by using western blotting with a Lat antibody (9166, Cell Signaling Technology) and clones lacking Lat eventually grown to an appropriate population for around 20 days.

### Sample Preparation

E6.1, JCaM1, P116, and Lat KO cells were cultured in RPMI media (Gibco) supplemented with 10% fetal calf serum (FCS) and transfected by electroporation (NEON, Invitrogen) to express WT and mutant Lck, Sr15, Lck10, and TSAd fused to EGFP, mCherry, or PS-CFP2. For ccRICS experiments, cells were activated on anti-CD3ϵ (16-0037, eBioscience) and anti-CD28 (16-0289, eBioscience) antibody-coated coverglass by allowing the cells to settle upon the activating surface for 10 min at 37°C prior to imaging. For PALM experiment, cells were activated for 10 min and subsequently fixed in 4% paraformaldehyde for 13 min. Antibody was adsorbed onto surfaces by incubating clean glass coverslips with 10 μg/ml antibody for at least 1 h at 37°C.

### Cross-Correlation Raster Image Correlation Spectroscopy

The ccRICS measurements were performed on a Zeiss LSM780 laser scanning microscope, using a LCI Plan-Neofluar NA = 1.3 water immersion 63× objective (Zeiss, Germany). Lck–GFP was excited with the 488-nm emission line of an Argon laser. Lck–mCherry was excited with the 561 nm emission line of a diode pump solid state (DPSS) laser. Lck–GFP and Lck–mCherry were measured simultaneously with GaAsP detectors using the 493–556-nm and 613–696-nm collection ranges, respectively. For each channel, the pinhole was set to 1 AU. For each ccRICS experiment, we acquired a stack of 100 frames in a selected field next to the cell edges. The pixel frame size of the image field was set to 256 × 256 and collected at an electronic zoom that resulted in a pixel size of 50 nm. The pixel dwell time was set to 12.61 μs/pixel, which resulted in a line time of 7.56 ms and frame time of 1.15 s. The acquired ccRICS data were processed and analyzed by the SimFCS software developed at the Laboratory for Fluorescence Dynamics (www.lfd.uci.edu) as described in the previously published papers (31, 32).

Briefly, for each two-color experiment, the RICS function was calculated in channels 1 and 2 for the entire image stack, with a moving average applied to remove slow cell movements. The resulting 3D RICS profile was then fit to a one-component diffusion model in each channel and the *G*(0) values and diffusion coefficients were derived from the fits recorded. The cross RICS function was then calculated between the two channels fit to a one-component diffusion model and the cross *G*(0) value and diffusion coefficient derived from the fit recorded. The fraction of molecules bound was then derived by taking the ratio of *G*(0)_CROSS_/*G*(0)_CH1_ if *G*(0)_CH1_ < *G*(0)_CH2_ or *G*(0)_CROSS_/*G*(0)_CH2_ if *G*(0)_CH2_ < *G*(0)_CH2_.

### PALM Imaging

Photoactivated localization microscopy images were acquired on a TIRF microscope (ELYRA; Zeiss) with a 100×, NA = 1.46 oil-immersion objective. For PS-CFP2, photoconversion was performed with 8 μW of 405-nm laser radiation and imaging of green-converted PS-CFP2 with15–30 mW of 488-nm light. For PALM, 15,000–20,000 images were acquired per sample using a cooled, electron-multiplying CCD (EMCCD) camera (iXon DU-897D, Andor) with an exposure time of 18 ms. Recorded images were analyzed using Zeiss ZEN software. Drifting of the sample during acquisition was corrected relative to the position of surface-immobilized 100 nm colloidal gold beads (BBInternational, UK) that were placed on each sample.

### PALM Data Processing

SMLM data were analyzed using custom software written in MATLAB (MathWorks) for detection of clusters and extraction of clustering parameters. Typically, for each cell, one to five non-overlapping representative regions of 4 μm × 4 μm were selected for analysis.

First, we used Ripley’s *K* function as previously described (44) to determine the extent of clustering of a population of molecules compared to a randomly distributed set of molecules. This was calculated using SpPack, an add-in for Microsoft Excel (45), as well as a custom MATLAB version optimized for larger data sets. In short, the Ripley’s *K* function calculates for each molecule the number of neighbor molecules within a given radius *r* corrected by the total density; finally, for each radius, the average is calculated over all molecules. The Ripley’s *K* function provides ensemble information on the whole region of interest; it provides information on the level of clustering of molecules in a region; however, no analysis is performed at the cluster level, and therefore, no information is available on individual clusters.

To retrieve information on individual clusters, we used density-based spatial clustering application with noise (DBSCAN) analysis (46) to identify individual clusters. The DBSCAN method detects clusters using a propagative method, which links points belonging to the same cluster based on two parameters: the minimum number of neighbors ϵ (ϵ = 3) in the radius *r* (*r* = 20 nm). The DBSCAN routine is implemented in MATLAB and subsequently coded in C++ and compiled in a Matlab executable (MEX) file to improve the speed of processing.

### Statistics

Statistical significance of the means of two data sets was assessed with unpaired *t*-test with Welch’s correction for the ccRICS data sets, which displayed normal distributions, and with a Mann–Whitney test for the PALM data sets, which did not all have normal distributions.

## Author Contributions

NK-K, FK, EC, and YY did and analyzed ccRICS and PALM experiments; EH conceived and analyzed ccRICS experiments; ZY and JL contributed to molecular biology; TT designed the analysis of PALM data; KG and SP contributed to the manuscript preparation; JR was responsible for conceptualization and the manuscript preparation.

## Conflict of Interest Statement

The authors declare that the research was conducted in the absence of any commercial or financial relationships that could be construed as a potential conflict of interest.
