# Supplementary material for: Distinct Mechanisms Regulate Lck Spatial Organization in Activated T Cells
Source: Front Immunol. 2016 Mar 8;7:83. doi: 10.3389/fimmu.2016.00083 (PMC4782156; doi:10.3389/fimmu.2016.00083)
Supplement: Supplementary file 1 [file Image_1.PDF]

Supplementary Material

Distinct mechanisms regulate Lck spatial organization in activated T cells

Natasha Kapoor-Kaushik, Elizabeth Hinde, Ewoud B. Compeer, Yui Yamamoto, Felix Kraus, Zhengmin Yang, Jieqiong Lu, Sophie V. Pageon, Thibault Tabarin, Katharina Gaus* and Jérémie Rossy*

*** Correspondence:** Jérémie Rossy and Katharina Gaus: j.rossy@unsw.edu.au, k.gaus@unsw.edu.au

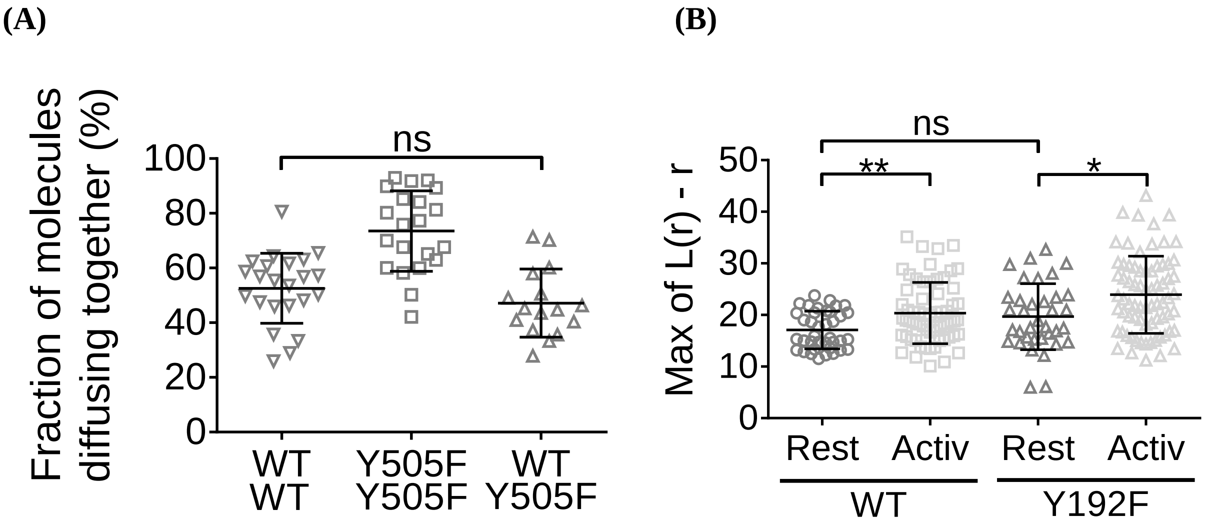

Supplementary Figure 1. (A) Open Lck has only a higher affinity for itself and not for WT Lck. JCam1 cells expressing (1) WT Lck–EGFP and WT Lck–mCherry, (2) constitutively open Lck(Y505F)–EGFP and Lck(Y505F)–mCherry or (3) WT Lck–mCherry and Lck(Y505F)–EGFP were activated and imaged as in Figure 1. Data for (1) and (2) are the same as plotted in figure 2. ns: non-significant. **(B) WT Lck and Lck(Y192F) have the same level of clustering in resting cells.** JCam1 cells transfected with either Lck WT or Lck(Y192F) labelled with PSCFP2were let to adhere on a surface coated with 5µg/ml anti-human CD90, fixed, imaged and analyzed as in Figure 1.
