# Supplementary material for: Distinct Mechanisms Regulate Lck Spatial Organization in Activated T Cells
Source: Front Immunol. 2016 Mar 8;7:83. doi: 10.3389/fimmu.2016.00083 (PMC4782156; doi:10.3389/fimmu.2016.00083)
Supplement: Supplementary file 2 [file Image_2.PDF]

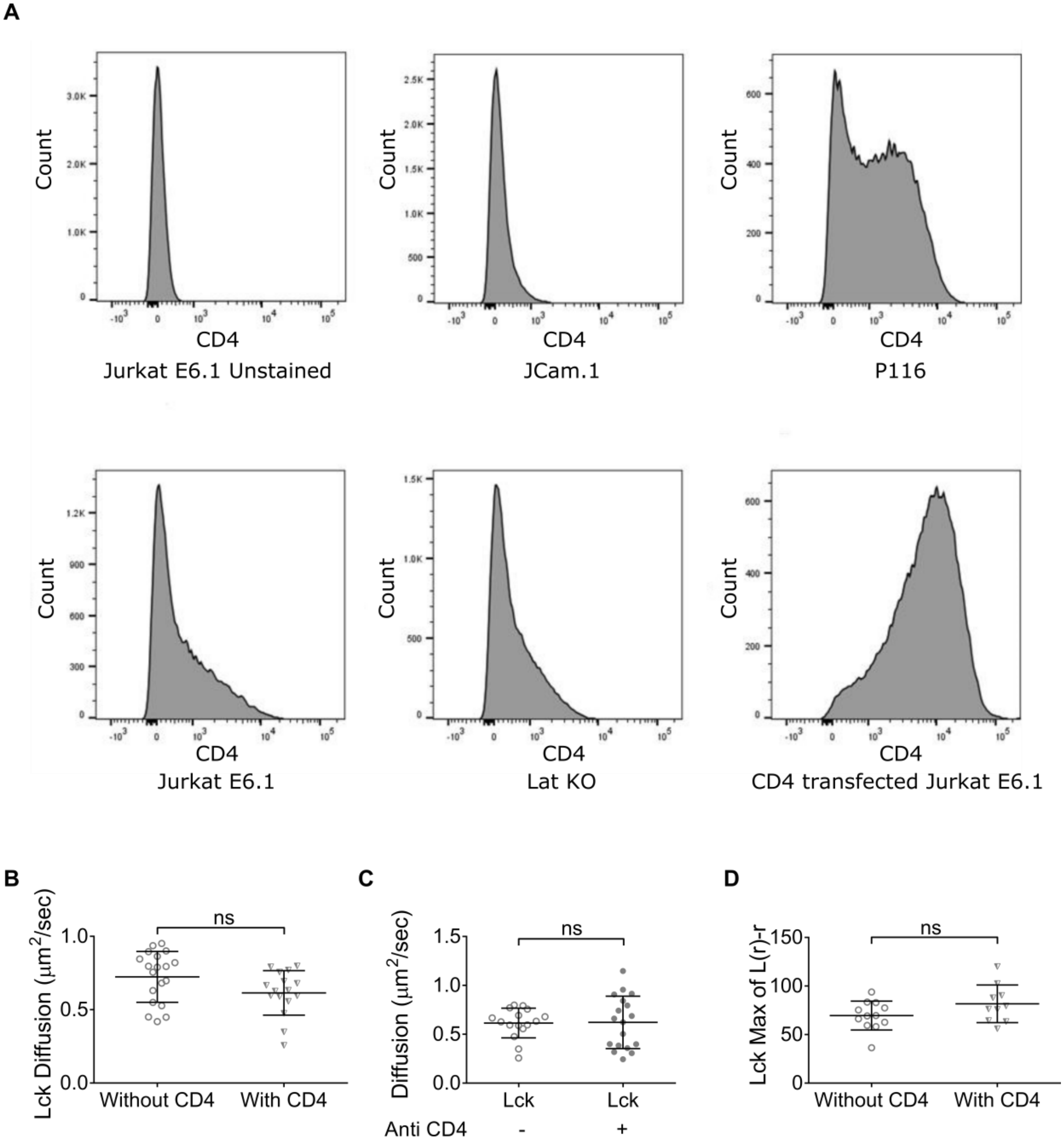

Supplementary Figure 2. CD4 does not contribute to Lck diffusion or clustering. (A) Analysis of CD4 expression in wild type Jurkat E6.1, JCam1, P116, Lat knock-out and E6.1 transiently transfected with CD4. For staining with CD4, 1x10^6 cells were incubated with 0.5ug/mL Alexa Fluor® 647 anti-CD4 (clone RPA-T4) on ice for 1 h. Flow cytometric analysis was performed using FACSCanto II (Becton Dickinson) and data analysis using FlowJo software. **(B)** Diffusion coefficient measured by RICS of Lck-EGFP in activated JCam1 cells transiently transfected or not with CD4 **(C)** Diffusion coefficient of Lck-EGFP in JCam1 co-expressing Lck and CD4 and activated on surfaces coated with antibodies against CD3, CD28 and ±CD4. **(D)** Clustering of Lck in activated JCam1 cells transiently transfected or not with CD4. Each symbol represents a cell. ns: non-significant. Data are from at least three independent experiments with a total of at least twelve cells.
