# Supplementary material for: Distinct Mechanisms Regulate Lck Spatial Organization in Activated T Cells
Source: Front Immunol. 2016 Mar 8;7:83. doi: 10.3389/fimmu.2016.00083 (PMC4782156; doi:10.3389/fimmu.2016.00083)
Supplement: Supplementary file 3 [file Image_3.PDF]

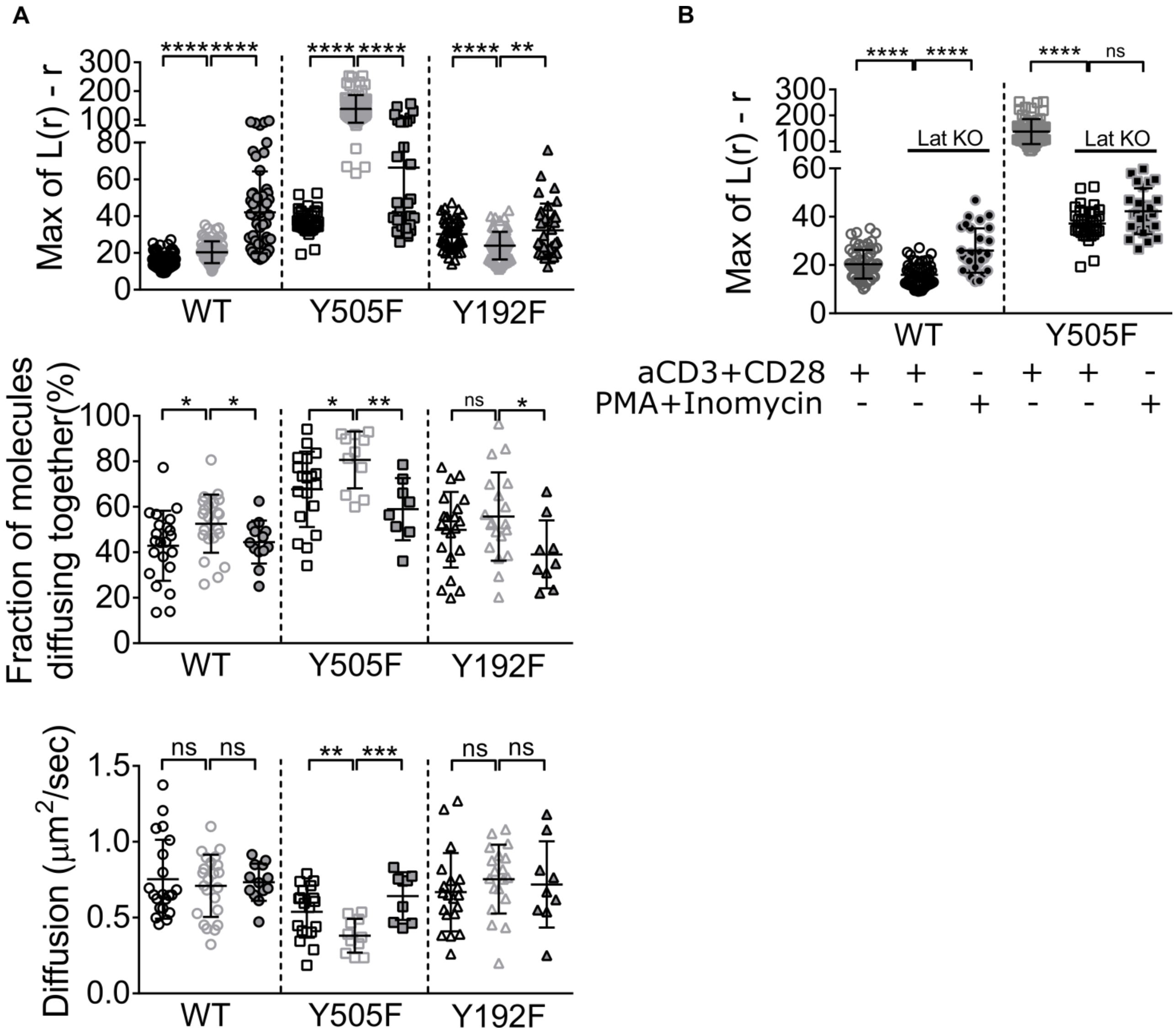

Supplementary Figure 3. Variation of Lck spatial organization in JCam1, Lat KO and P116 cells (A) Comparison of the clustering (top), propensity to aggregate (middle) and diffusion coefficient (bottom) of (1) WT Lck, (2) Lck(Y505F) and (3) Lck(Y192F) between Lat KO cells (black symbols), JCam1 cells (grey symbols) and P116 cells (black line, grey filling symbols). Data are the same than the data plotted in Figure 2, 3 and 4. **(B)** Clustering of Lck in cells expressing or lacking Lat and that have been stimulated with antibodies against CD3 and CD28 or with PMA-ionomycine. Lat-KO cells were incubated for 20 min at 37˚C on non-activating glass surfaces followed by 10 min incubation with or without 50 ng/ml Phorbol 12-myristate 13-acetate (P1585, Sigma-Aldrich) and 1 µM ionomycin (I3909; Sigma-Aldrich). Data from JCam1 and Lat KO cells activated by antiCD3+28 are from figure 2 and 4. *P < 0.05, **P < 0.005 , ***P < 0.0005 and ****P < 0.00005 (unpaired t-test and Mann-Whitney tests). Data are from three to five independent experiments with a total of at least nineteen cells.
