# Supplementary material for: Distinct Mechanisms Regulate Lck Spatial Organization in Activated T Cells
Source: Front Immunol. 2016 Mar 8;7:83. doi: 10.3389/fimmu.2016.00083 (PMC4782156; doi:10.3389/fimmu.2016.00083)
Supplement: Supplementary file 4 [file Image_4.PDF]

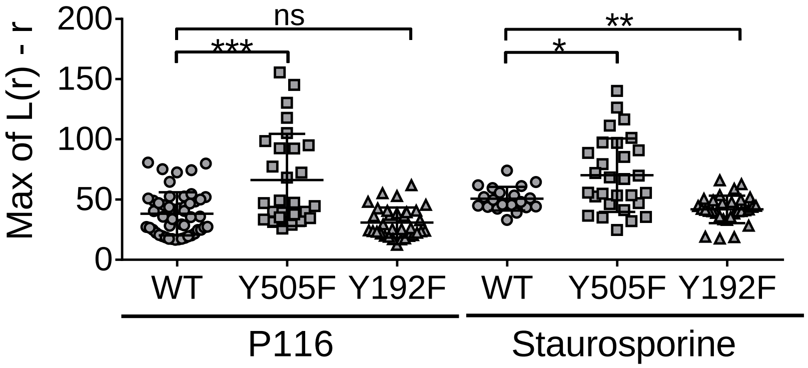

Supplementary Figure 4. Staurosporine had similar effect on Lck clustering as the absence of Zap-70. Comparison of the clustering of (1) WT Lck, (2) Lck(Y505F) and Lck(Y192F) between Zap70 deficient cells P116 and JCam1 cells treated with a pan-kinase inhibitor Staurosporine. JCam1 cells were pre-treated for 30 min with 10nM staurosporine (S5921; Sigma-Aldrich) before 10 min incubation with activating glass surfaces at 37˚C *P < 0.05, **P < 0.005 , **P < 0.005 and ***P < 0.0005 (unpaired t-test). Data are from three to five independent experiments with a total of at least fifteen cells.
